# White-naped mangabeys’ viable insurance population within European Zoo Network

**DOI:** 10.1038/s41598-020-80281-6

**Published:** 2021-01-12

**Authors:** Carlos Iglesias Pastrana, Francisco Javier Navas González, María Josefa Ruiz Aguilera, José Antonio Dávila García, Juan Vicente Delgado Bermejo, María Teresa Abelló

**Affiliations:** 1 University of Córdoba, Córdoba, Spain; 2 Department of Conservation, Córdoba Zoo Park, Córdoba, Spain; 3 Wildlife Resources Research Institute, Ciudad Real, Spain; 4 White-naped mangabey EEP Coordination (EAZA: European Association of Zoos & Aquariums), Parc Zoològic de Barcelona, Barcelona, Spain

**Keywords:** Genetics, Animal breeding

## Abstract

The success and viability of an ex-situ conservation program lie in the establishment and potential maintenance of a demographically and genetically viable insurance population. Such population reserve may support reintroduction and reinforcement activities of wild populations. White-naped mangabeys are endangered restricted-range African primates which have experienced a dramatic population decrease in their natural habitats over the last few decades. Since 2001, some European zoos singularly monitor an ex-situ population aiming to seek the recovery of the current wild population. The aim of the present paper is to evaluate the genetic status and population demographics of European zoo-captive white-naped mangabeys based on pedigree data. The captive population is gradually growing and preserves specific reproductive and demographic parameters linked to the species. The intensive management program that is implemented has brought about the minimization of inbreeding and average relatedness levels, thus maintaining high levels of genetic diversity despite the existence of fragmented populations. This finding suggests white-naped mangabey ex-situ preservation actions may be a good example of multifaceted conservation throughout studbook management which could be used as a model for other ex-situ live-animal populations.

## Introduction

The Intergovernmental Science-Policy Platform on Biodiversity and Ecosystem Services (IPBES) warned about the high risk of extinction of over one million animal and plant species in 2019^1^. To counteract this global extinction crisis, ex-situ conservation programmes have been increasingly implemented, seeking the sustainable maintenance and breeding of threatened species under controlled conditions outside their natural habitat.

Strategically combined with reintroduction activities, ex-situ techniques have become effective measures to preserve endangered species, when the efficient preservation of wild populations is compromised^2–4^. Integral management of captive-bred populations implies genetic and demographic routine monitoring tasks must be performed to reach conservation objectives in these captive populations^4^.

Monitoring tasks seek to ensure that genetic diversity and effective population sizes are maintained within acceptable levels, while inbreeding and average relatedness between mating animals are reduced to a minimum^5,6^. High levels of genetic diversity do not necessarily imply a high heritability of features that may be desirable from a conservation perspective. Evolutionary potential depends on external, and often complex, components for example, the variability that is due to environment. Provided evolutionary potential^7^ is thought to be partially driven by genetic diversity^8^, it is the effectiveness of genetic management policies (environmental factors even if this are induced by humans), which may determine whether long-term population viability and reintroductions into the wild are successfully maximized.

The European Association of Zoos and Aquaria (EAZA) gathers together the leading zoos and aquariums in Europe and the Middle East and regularly designs standards for the conservation of nature and wildlife both at its member institutions (400 member zoos and aquariums across 48 countries) and outside the zoo premises^9^. Under the scope of the Zoos European Directive^10^, EAZA member institutions are instructed to maintain standardized individualized records to make the interpretation and utilization of animal databases easily accessible for all the members involved in cooperative management plans and formal research projects^11^. Internally, these databases allow animal management staff to plan and monitor population conservation and care programmes for long-term survival and potential in-situ conservation assistance^12^.

Updated records of zoo collections are a valuable source of information for scientific researchers pursuing to reach the key mission conservation goals of zoos and aquaria as stated in the first set of guidelines of the World Association of Zoos and Aquariums^13^. To ensure these goals are accomplished, each individual animal record should comprise all the relevant data concerning its current status, origin, genealogy, possible transactions, health and other practical advice (i.e. welfare issues and/or behavioural features)^14^.

The documentation of the complete historical record of every individual lays the basis for better coordinated genetic and demographic population management practices in threatened taxa^15^. This information may not only enhance the ability to adapt housing and living conditions to approximate captivity to wild environmental conditions, but also to replicate the genetic interaction of the individuals in this recreated environment^16^.

Pedigree information analysis constitutes an invaluable tool for genetic diversity quantification and demographic structure evaluation in captive populations. This information can be used to formulate recommendations to maintain a genetically-healthy population^17^ with reliable reproduction and increasing group growth rates^18^. Such recommendations may involve translocations among zoological nuclei, and the objective selection of most appropriate individuals for reproduction and/or identification of related animals^19^.

Pedigree-based strategies are cost-effective alternatives to perform routine genetic diversity evaluations, population demographics and viability and to track the improvement of genetic diversity^16,20^. The effectiveness of pedigree analyses relies on the strict control of genealogical information carried in endangered populations, from the moment when the base captive-founder populations were established. The integrity of the genealogical information present in pedigrees may be compromised by problems associated to the veracity and effectivity of the tools used^21–23^, pedigree completeness levels ^24,25^ and of the thoroughness of the operators participating in the process of data collection and registration^21^, among others. The estimates derived from the analyses of non-robust pedigrees (i.e., low depth, missing information, errors, unknown founder relationships, among others) can be favoured if empirical estimates of relatedness via genetic markers (microsatellites or SNPs) are determined^26^. Hence, endeavouring to improve pedigree robustness may always be sought to improve the accuracy of genetic parameters^27^.

In this context, budget limitations^28,29^ may frequently compromise the routinely application of genomic tools and restrict their utility to small-sized threatened populations, with limited or missing genealogical background, in which the proportion of polymorphic loci is commonly small^19,30,31^. Otherwise the use of large numbers of genomic markers may be needed^32^. These limitations may result in allele frequencies of the historical population being unknown, which may bias the inference of inbreeding as a direct consequence of potential changes occurring due to genetic drift^33^. As a result, molecular techniques, which may not distinguish between identity by descent (IBD) and identity by state (IBS) probabilities underlying genetically mediated similarities among relatives^34,35^, may compliment the information comprised in pedigrees.

Based on the aforementioned comparison, *ex-situ* management programmes using pedigrees are routinely carried out by EAZA in a wide range of animal species. These species mainly consist of terrestrial and marine mammals (approximately 26% of threatened species at a global level)^15^. In the case of non-human primates, nearly 60% of the species are endangered and 75% account for wild reduced populations as a result of human-induced disturbs during the last three decades^36^.

Among other genus and families, EAZA institutions host the largest captive population of white-naped mangabeys (*Cercocebus atys lunulatus*), a West African endemic non-human primate from (Ghana, Republic of Côte d'Ivoire and Burkina Faso)^37^ currently classified as ‘Endangered’ by The Red List of IUCN due to habitat fragmentation and bushmeat^38^. The genealogical information (ESB, European Studbook) of captive white-naped mangabeys^19^ has been monitored in different European zoos since 1994.

Since 2000, a European Endangered Species Programme (EEP) was implemented to respond to the classification of the species as ‘Critically Endangered’ by IUCN^19^. Supported by the West African Primate Conservation Action (WAPCA) in Ghana and Côte d’Ivoire since 2010, the first research outcomes obtained consisted of a population viability analysis (PVA) simulating different scenarios combining deterministic and stochastic factors potentially affecting white-naped mangabey wild populations’ dynamics. The study concluded genetic diversity may remain high under all assumptions (> 90%)^19^, which suggested European captive mangabeys may act as an insurance population to accomplish *in-situ* conservation goals.

The present study evaluates the effectiveness of conservation activities along the history of the captive population of white-naped mangabeys. The genetic and demographic structure of the captive population, genetic parameters and the trends described by them were quantified. Afterwards, a breeding strategy is proposed to recommend the most appropriate animal matings among the individuals present in the current population. To conclude, the genetic distances among hosting institutions were quantified and traced. The present set of analyses may guide future conservation aimed breeding strategies and act as a model for other species under the similar circumstances.

## Results

### Intensive management policies drive demographic rising

**Figure 1:**
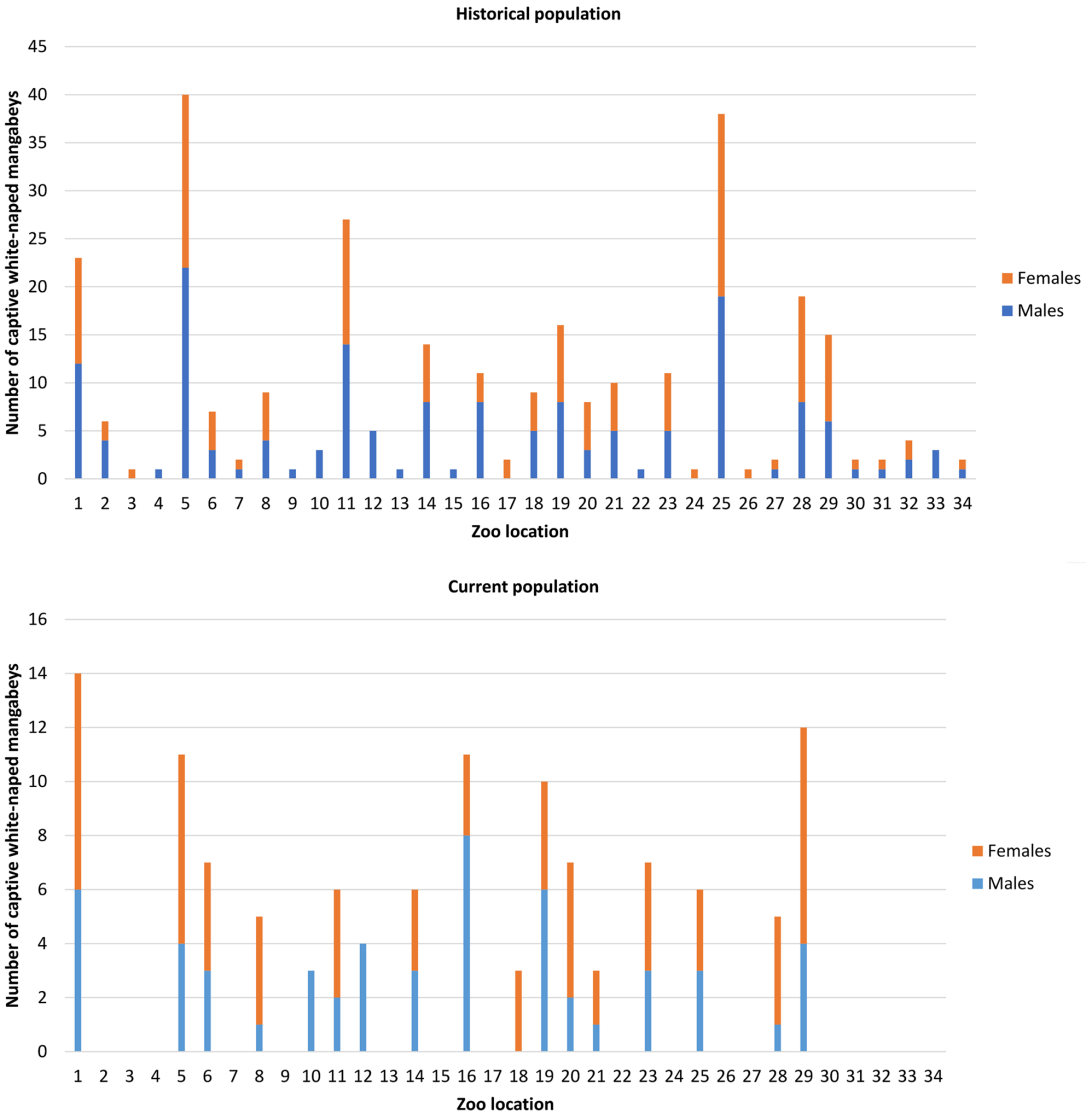
Historical and current distribution of individuals across institutions.

**Figure 2:**
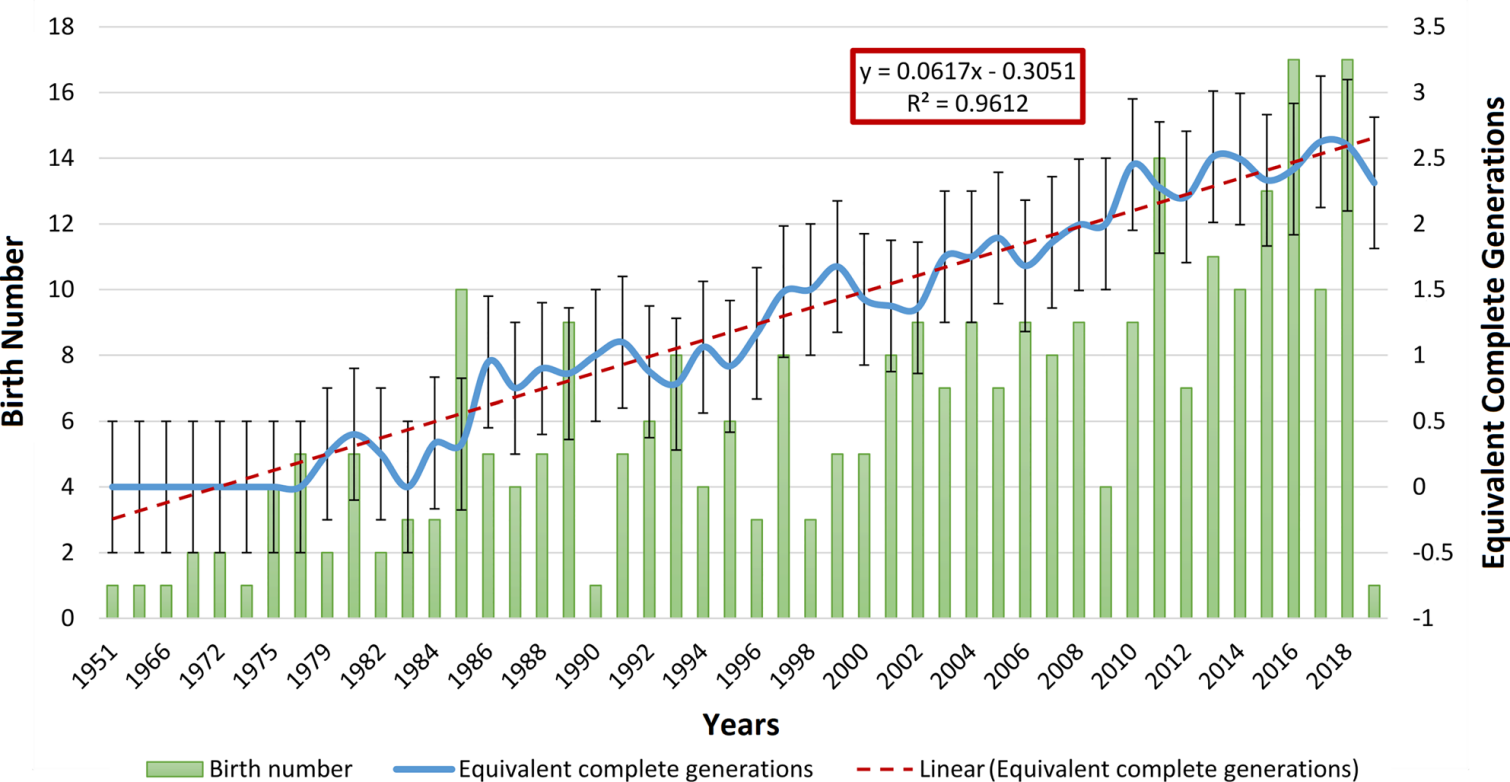
Evolution of birth number and equivalent complete generations in the historic population (n = 298) from 1951 to 2019. Provided we measured the variability of time-series data, we relied on the standard error of the mean (SEM) rather than the standard deviation (SD), as it removes variability imposed by the trend in the data, which the SD does not.

The historical and current distribution of individuals across institutions is shown in Fig. 1. The average (± SD) number of infants born per year in the historic population was 6.20 ± 4.10, reaching its peak (17) in 2016 and 2018. The average (± SD) number of complete equivalent generations during the last decade (2008–2018) was 2.39 ± 0.218, and described a linear increasing tendency until it reached a maximum value of 2.59 in 2018 (Fig. 2).

Pedigree completeness indexes (PCIs) for one, two, three, four, five and six generations, the maximum number of traced generations, maximum number of complete generations and number of equivalent generations in the two population sets, are shown in Table 1.

**Table 1:** Summary of statistics of genealogy, demographic and offspring analysis in the historical and current populations of white-naped mangabey.

Parameter		Population set	
		Historical	Current
Population size		298	120
Genealogy analysis	Maximum number of traced generations, n	6	6
	Pedigree completeness level at 1st generation, (Known parents)	82.38	92.08
	Pedigree completeness level at 2nd generation, (Known grandparents)	52.43	66.45
	Pedigree completeness level at 3rd generation, (Known great grandparents)	20.72	32.91
	Pedigree completeness level at 4th generation, (Known great great grandparents)	4.19	7.65
	Pedigree completeness level at 5th generation, (Known great great great grandparents)	0.29	0.67
	Pedigree completeness level at 6th generation, (Known great great great great grandparents)	0.000052	0.01
	Mean number of maximum generations (± SD)	2.24 ± 1.51	2.95 ± 1.51
	Mean number of complete generations (± SD)	1.28 ± 0.85	1.56 ± 0.76
	Mean number of equivalent generations (± SD)	1.60 ± 0.97	2.00 ± 0.89
Demographic and offspring analysis	Males%	52.68	45.00
	Mean (± SD) number of infants per male, n	1.57 ± 4.17	2.07 ± 4.19
	Maximum infant number per male, n	27	20
	Average age of males in reproduction, years	16.10	15.22
	Females%	47.31	55.00
	Mean (± SD) number of infants per females, n	1.79 ± 3.02	1.92 ± 2.78
	Maximum infant number per female, n	15	10
	Average age of females in reproduction, years	16.57	14.00
	Female/male ratio	0.90/1	1.22/1
	Progeny from males selected for breeding, %	45.62	84.10
	Progeny from females selected for breeding, %	41.34	75.73

The maximum progeny per male and female decreased almost linearly in the current population. However, the mean (± SD) progeny per male was 1.57 ± 4.17 in the historic population and 2.07 ± 4.19 in the current population; 1.68 ± 3.02 and 1.92 ± 2.78 in the historic and current population, respectively, for females. The female/male ratio is increased in the current population (1.22/1) in respect of the historic population (0.90/1). The percentage of males with progeny selected for breeding, that is all males whose offspring has acted as a breeding male or female, was 45.62% and 84.10% in the historic and current population, respectively; 41.34% and 75.73% for females, all females whose offspring has acted as a breeding female or male (Table 1). The average generation interval and the mean age of parents at offspring’s birth and dispersion statistics (SD and SEM) are presented in Table 2, respectively.

**Table 2:** Average generational intervals and mean age of the parents at the birth of their offspring (years) and dispersion statistics (Standard deviation, SD and Standard error of the mean, SEM) for population groups.

Population set	Parameter					
	Generation interval route	Sire to son	Dam to son	Sire to daughter	Dam to daughter	Total
Historical (n = 298)	n	23	22	39	36	120
	Mean	15.07	11.28	14.73	11.28	13.13
	SD	5.25	4.55	5.16	4.42	5.12
	SEM	1.09	0.97	0.82	0.73	0.46
Current (n = 120)	N	14	14	30	30	88
	Mean	13.98	10.62	15.34	11.57	13.08
	SD	5.76	4.58	5.27	4.71	5.33
	SEM	1.54	1.22	1.41	1.26	0.57

Population set	Parameter					
	Age of the parents at the birth of their offspring	Sire to son	Dam to son	Sire to daughter	Dam to daughter	Total
Historical (n = 298)	n	134	132	113	112	491
	Mean	14.93	11.74	14.75	10.96	13.12
	SD	4.70	4.84	5.05	4.85	5.15
	SEM	0.41	0.42	0.47	0.46	0.23
Current (n = 120)	N	48	48	62	63	221
	Mean	14.17	9.99	14.51	10.20	12.23
	SD	4.92	3.78	5.13	4.69	5.13
	SEM	0.71	0.55	0.74	0.68	0.35

### Identity by descent estimators and degree of non-random mating

Although mean (± SD) inbreeding is low (3.19% ± 0.07% in the historic population and 1.64% ± 0.05%) in the current population), highly inbred animals are present in each population set (12.75% and 4.16% of the animals in the historic and current population, respectively).

The percentage of inbred animals was 23.15% and 17.5%; the average (± SD) coancestry was 4.21% ± 2.00% and 4.18% ± 2.00%; and the degree of non-random mating presented mean values of − 0.02 ± 0.07 and − 0.03 ± 0.05, for the two population sets, respectively. The highest values for these three parameters were 0.25 (25%) for different ages between 1985 and 2018, 0.0914 (9.14%) for coancestry in 1997 and 0.225 for non-random mating degree in 1999.

Matings resulting in highly inbred animals have occurred in the population: 2 (0.67%) mating between full sibs, 18 (6.04%) mating between half-sibs y 18 (6.04%) mating between parent-offspring.

Registered values for mean (± SD) Genetic Conservation Index (GCI) were of 2.84 ± 1.48 and 3.52 ± 1.52 for the historic and current population, respectively. The summary of identity by descent estimators, non-random mating degree and genetic conservation index parameters is presented in Table 3.

**Table 3:** Statistics of identity by descent estimators, non-random mating degree and genetic conservation index.

Parameter	Populational sets	
	Historical (n = 298)	Current (n = 120)
Inbreeding (F, %)	3.19 ± 0.07	1.64 ± 0.05
Average (± SD) individual increase in inbreeding (ΔF, %)	5.53 ± 0.16	1.54 ± 0.07
Maximum coefficient of inbreeding (%)	25.00	25.00
Inbred animals (%)	23.15	17.5
Highly inbred animals (%)	12.75	4.16
Average (± SD) coancestry (C, %)	4.21 ± 2.00	4.18 ± 2.00
Average (± SD) relatedness (ΔR, %)	8.43 ± 4.79	8.36
Average (± SD) Non-random mating rate (α)	− 0.02 ± 0.07	− 0.03 ± 0.05
Average (± SD) Genetic Conservation index (GCI)	2.84 ± 1.48	3.52 ± 1.52

**Figure 3:**
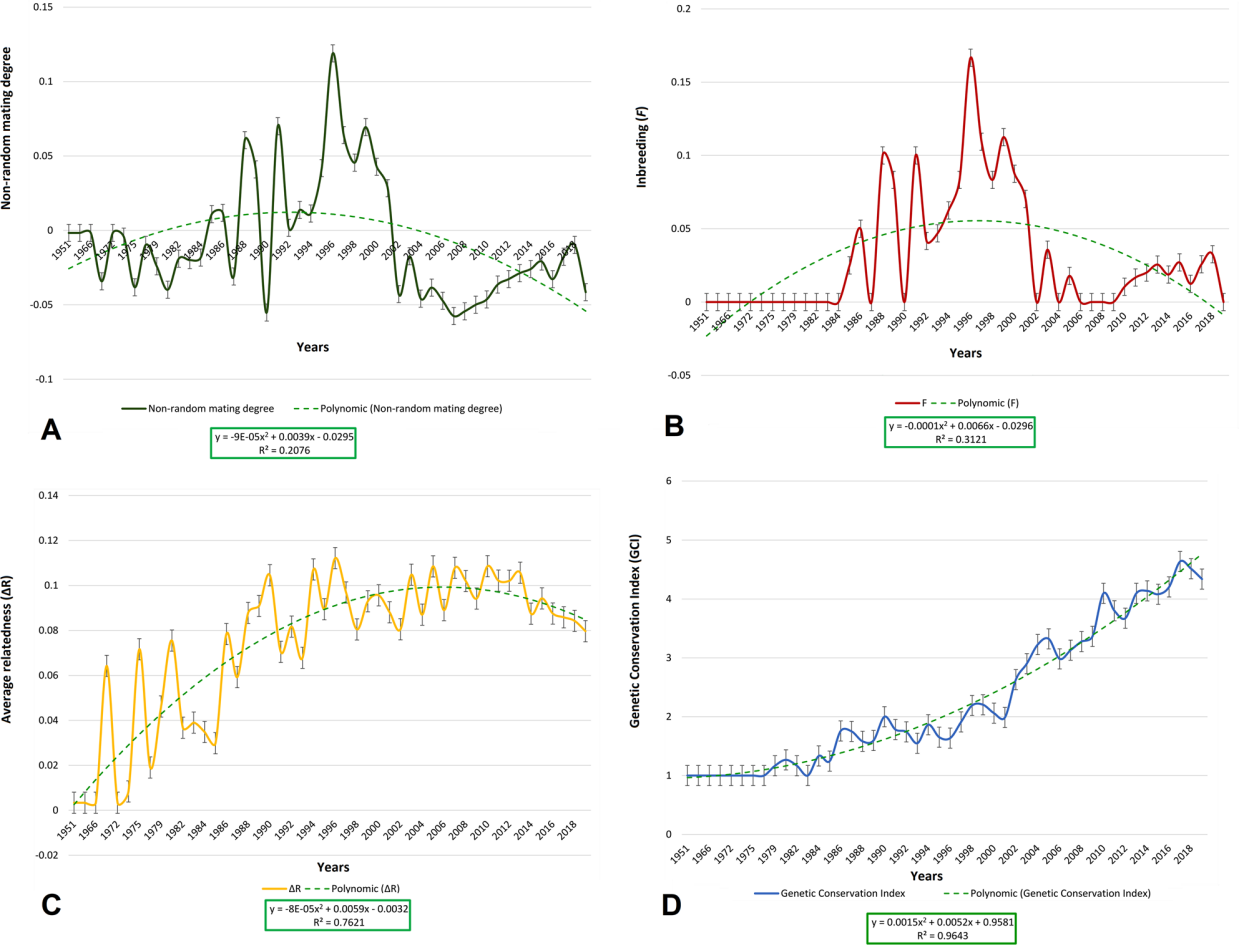
Evolution of (**A**) non-randon mating degree (*α*), (**B**) inbreeding rate (*F*), (**C**) average relatedness (Δ*R*) and (**D**) Genetic Conservation Index (ICG) for the white-naped mangabey captive population from 1951 to 2019. Provided we measured the variability of time-series data, we relied on the standard error of the mean (SEM) rather than the standard deviation (SD), as it removes variability imposed by the trend in the data, which the SD does not.

**Figure 4:**
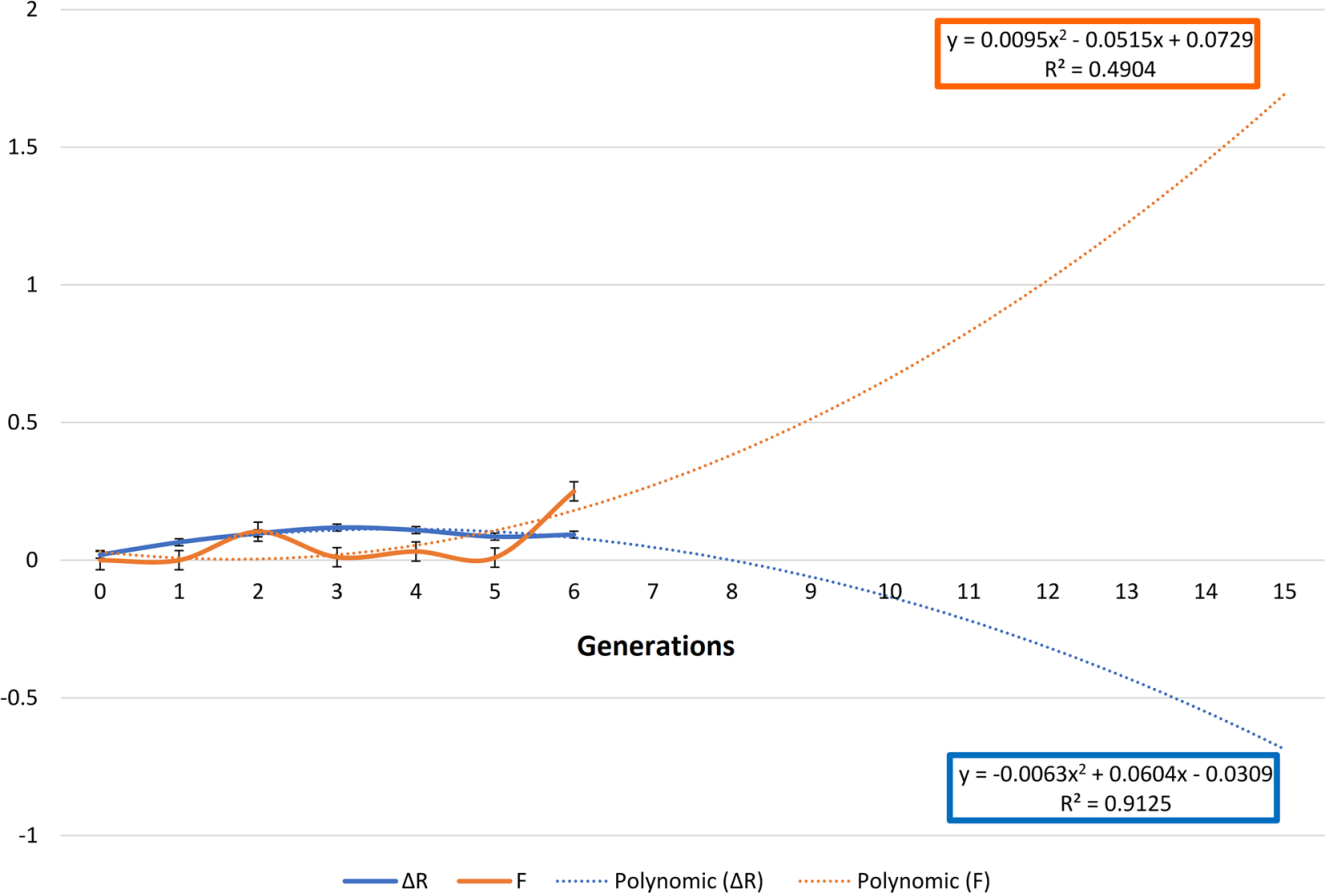
Regression equations for average inbreeding (*F*) and average relatedness (Δ*R*) expressed in % from 1ª to 6ª generation, and their prediction from 7ª to 15ª generation. Provided we measured the variability of time-series data, we relied on the standard error of the mean (SEM) rather than the standard deviation (SD), as it removes variability imposed by the trend in the data, which the SD does not.

The evolution of the non-random mating degree (*α*), inbreeding rate (*F*), average relatedness (Δ*R*), and Genetic Conservation Index (GCI) of the European captive white-naped mangabey from 1951 to 2019 is represented in Fig. 3. Regression equations for the prediction of the evolution of average inbreeding (*F*) and average relatedness (Δ*R*) up to 15 generations are shown in Fig. 4. Linear, logarithmic and polynomic functions were tested seeking the best fitting models to describe the trends presented by each parameter. The polynomic function was selected upon considering the functions reporting the highest value for the determination coefficient (R^2^)^39^.

### Probabilities of gene origin, ancestral contributions and genetic diversity

The results for the analysis of the gene origin probabilities, ancestral contributions and genetic diversity, are shown in Table 4.

**Table 4:** Summary of results for founder analysis, measures of genetic diversity and diversity loss.

Parameter	Reference population (both parents known historically) (n = 240)
Historical population	298
Current population	120
Base population (one or more unknown parents)	58
Actual base population (one unknown parent = half founder)	11
Number of founders, n	29
Number of ancestors, n	33
Effective number of non-founders (N_*ef*_)	51.32
Number of founder equivalents (*f*_*e*_)	15.41
Effective number of ancestors (*f*_*a*_)	11
Founder genome equivalents (*f*_*g*_)	11.85
*f*_*a*_/*f*_*e*_ ratio	0.71
*f*_*g*_/*f*_*e*_ ratio	0.77
Genetic diversity, GD (%)	95.78
Genetic diversity in the reference population considered to compute the genetic diversity loss due to the unequal contribution of founders, GD (%)	96.75
GDL due to bottlenecks and genetic drift since founders (GL) (%)	4.22
GDL due to unequal founder contributions (%)	3.24
GDL due to genetic drift (%)	0.97
Ancestors explaining 25% of the gene pool (n)	1
Ancestors explaining 50% of the gene pool (n)	5
Ancestors explaining 75% of the gene pool (n)	10

*GDL* Genetic diversity loss.

Considering the marginal genetic contribution, the genetic constitution of a single ancestor (identification code: 33) explained 16.99% of the total genetic pool within the population (91.62%), 1.69% of the total inbreeding coefficient (3.09%) and 1.68% of the total coancestry (3.93%). The 10 ancestors with higher marginal genetic contributions were responsible for the total inbreeding and 3.72% of the total coancestry in the population.

The mean (± SD) effective population size calculated by the individual inbreeding rate was 47.33 ± 21.04 in the reference population, whereas the mean (± SD) effective population size based on the individual coancestry rate (*N*_*e*_*C*_*i*_) was 17.76 ± 1.59. The number of equivalent subpopulations (± SD) was 0.37 ± 0.17.

### Herd relationships and breeding strategy

The mean (± SD) number of animals per zoo was 8.51 ± 10.26, ranging from 1 to 40. Related to Wright’s F statistics, the inbreeding coefficient relative to the total population^40^ was − 0.01, the inbreeding coefficient relative to the subpopulation^41^ was − 0.16 and the correlation between randomly drawn gametes from the subpopulation relative to the total population (F_ST_) was 0.13 (Table 5). There were considered a total of 561 Nei’s genetic distance between the 35 zoos. The average (± SD) Nei’s genetic distance was 0.253 ± 0.126. The mean (± SD) coancestry within subpopulations was 0.16 ± 0.02 (16.00% ± 2.00%) and the mean inbreeding was 0.032 ± 0.02 (3.20% ± 2.00%). In the metapopulation, the mean (± SD) coancestry and self-coancestry were 0.04 ± 0.02 and 0.52, respectively.

**Table 5:** Wright’s Fixation statistics and zoo’s genetic distancing parameters.

Parameter	Value
F_IS_ (inbreeding coefficient relative to the Subpopulation)	− 0.16
F_ST_ (Correlation between random gametes drawn from the subpopulation relative to the total population)	0.13
F_IT_ (inbreeding coefficient relative to the total population)	− 0.01
Mean (± SD) number of animals per subpopulation	8.51 ± 10.26
Number of genetic Nei distances	561
Average (± SD) Nei genetic distance	0.25 ± 0.13
Mean (± SD) coancestry within subpopulations	0.16 ± 0.02
Selfcoancestry	0.52
Mean (± SD) coancestry in the metapopulation	0.04 ± 0.02
Subpopulations	35

**Figure 5:**
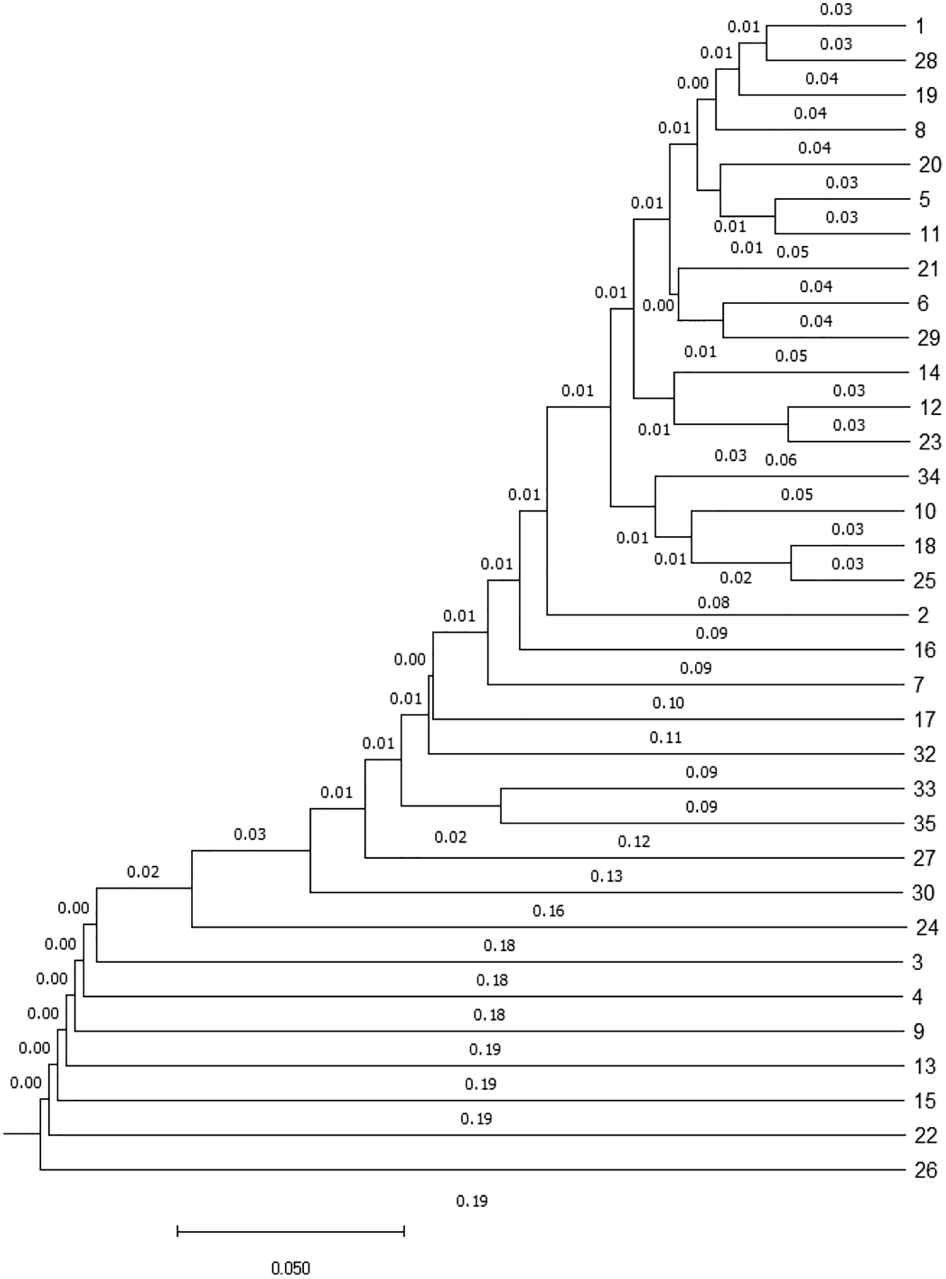
Cladogram constructed from Nei’s genetic distances among EAZA member institutions that house white-naped mangabeys in their facilities.

Zoo structure assessment revealed none of the zoos could be considered the population nucleus neither totally isolated. The number of zoos that used foreign father was 19, whereas 17 used own fathers and 14 used both foreign and own fathers. In total, 28 pairs of zoos showed the greatest Nei’s genetic distance (50%) among them. The minimum Nei’s genetic distance was 0.0619 (6.19%) and was shared between one pair of zoos (Supplementary Table S1). A cladogram representing all the relationships between the 34 zoos is shown in Fig. 5.

## Discussion

Conservation measurements implemented in captive European white-naped mangabeys have focused on maintaining a healthy ex-situ population mimicking the natural framework of the species. The captive population has maintained high genetic diversity and minimized inbreeding levels since 1951 (see Fig. 3). Two of the highest annual birth rates were experienced in 2016 and 2018 (see Fig. 2)^4^. The minimum number of animals born per year (≤ 4) describes a cyclical trend which lasts approximately 20 years, a period after which white-naped mangabeys naturally display signs of reproductive senescence^42^.

The demographic evolution of captive population is parallel to the improvement of the risk situation faced by white-naped mangabeys wild populations, which promoted the reclassification of the species by The Red List of IUCN to the lower threat category of “Endangered” in 2015. The inclusion of twenty-one new institutions reinforced EAZA’s network and brought about the addition of new founders (twenty-eight unrelated, wild-born individuals) and genealogy-known individuals to the *ex-situ* population since 2012^19^. The progressive increase in pedigree knowledge up to 90% at first generation occurred in the context of the scarcely available data from other threatened species held at offsite emplacements for similar conservation purposes (for instance, 27%-35% known pedigree for sable antelope^32,43^ and 70.9% for African Penguin^44^).

This lack of information occurs even if institutions make every effort to implement the most efficient standardized methods. Pedigrees can remain problematic due to multiple reasons, including difficulties associated with discerning parentage with herd or flock breeding^22,23^, low generation depth^24^, unknown founder relationships^25^, and human error^21^, among others^45^.

As a result, the analysis of incomplete pedigree records may lead to biased calculations of demographic and genetic parameters, even if self-sustainability could be expected from most managed populations^46^. For instance, 78% of bird and 52% of mammal captive populations registered in EAZA’s studbooks^47^, have pedigree completeness index levels below 85% and 58% fail to achieve the target conditions for sustainability (effective population size, growth rates, sex ratio and similar life-time family sizes across zoos)^46^.

The pedigree completeness levels in our study provide the first evidence of the success of white-naped mangabey ex-situ programme, which in turn enhances the possibilities for protection and recovery at medium and long-term, as long as genealogical recording and intensive management husbandry practices continue^19^.

The captive population constitutes itself a short-term backup reserve if the imminent extinction of wild populations occurred. In fact, it is the intensive management implemented, which aims at preserving the demographic and biologic structure of white-naped mangabeys wild counterparts, which potentializes the breeding capacities of the individuals to effectively retain high levels of genetic diversity. Maximum progeny per male and female were higher in the historic population. However, this could be ascribed to the fact that in the origin of the captive population, the main objective was to ensure a number of animals which may permit the captive population’s long-term viability^48^.

High mean progeny per male and female in current population denote the balance of the differential contribution of individuals to reproduction may have effectively contributed to the maintenance of genetic diversity^49^. This was supported by the negative values of F_IS_ (Table 5), suggesting breeding policies implemented may enable maximizing the likelihood of unrelated matings pairs^50^.

Mean age of animals at breeding (Table 1) and mean age of parents at the birth of their offspring selected for breeding (Table 2) were lower in the current population. This may be indicative of the attempts to maximize reproductive potential promoting maternal reproductive skills and interactions during high fertility periods^42^. In these regards, feeding and handling in early growth stages, first parturition and lactation must be appropriate to ensure reproductive success is not affected^51^.

Prolonging generational intervals can effectively increase the number of animals selected for breeding, progressively increasing effective population sizes and, therefore, generating a proportional reduction in inbreeding^52^, which maximizes the preservation of genetic diversity. To increase selection pressure, older animals with more progeny registries may be required, which may extend generation intervals. Shortening generation intervals may imply younger animals with fewer progeny registries may be considered, which may decrease selection pressure. This negative correlation could be compensated as young animals often present a greater genetic value provided they are the result from maximized genealogical diversity practices^53^. By contrast, reduced generation intervals may imply a larger number of animals reach sexual maturity age (set around 6 years old) earlier, with the consequent increase in birth number and population growth rate.

Additionally, the balance between such strategies may lead to the success of the breeding programme, as suggested by the relatively low negative values of F_IS_, which may be indicative of the promotion of breeding policies that consider unrelated animals at a rate at which the population does not excessively depart from Hardy–Weinberg equilibrium.

Mean generation intervals were higher for the current sire-daughter and dam-daughter pathways, which could be explained by a sex-ratio which favours females (Table 1), a common demographic feature to wild populations of the genus *Cercocebus*^54^ which could be ascribed to the philopatric nature of primate females.

This biological condition provides primates with an important functional role for ecosystem health and wellbeing which simultaneously enables the survival and success of offspring to breed^55^. Additionally, in Old World monkeys (Family *Cercopithecidae*), milk amount and quality may be influenced by offspring sex, to the detriment of baby females^56,57^, which determines a lower growth rate in their early stages and a delay in the mean age of prepuberal females in reproduction.

Parents’ mean age at their offspring’s birth was slightly lower than generation intervals (Table 2), suggesting selection of breeding animals whose offspring may potentially breed is performed slightly later than the moment when their first offspring is born. This way, data and reproductive records (health status, sexual cyclicity and maternal skills) may adjust to life expectancy (males: 26.7 years; females: 34.7 years^58^) and maximum periods of fertility (males: 19 years; females: 15 years^19^) of captive individuals.

Historical and current percentages of females with progeny selected for breeding were lower than those of males (also slightly older). This suggests breeding selection policies pay a greater attention to males [either phenotypically (for instance, considering their behaviour, adaptability to environment or resistance to stress^58^), functionally (reproductive effectiveness) or conservationally (higher levels of genetic diversity and reduced inbreeding)] as suggested for other species^59^. This policies simulate the sexual dispersion of this species, in which the males constitute the migratory sex^60^.

The implementation of a EEP since 2000 significantly contributed to inbreeding and average relatedness reduction^19^. However, levels above 1% and highly inbred animals can be found in the captive population (Table 3), suggesting matings between closely-related animals may still occur. Although inbred individuals could be outcrossed with unrelated individuals from the wild^61^, obtaining new founders from wild populations is difficult, provided these populations currently describe a decreasing trend.

Inbreeding has remained below coancestry levels, suggesting matings among closely-related individuals were unintentionally performed^62^. The different subpopulations are substantially separated, making it difficult to involve different genetic resources. This is consistent with the degree of non-random mating (α) and F_IS_ values, which suggested higher rates of random matings among closely-related individuals may occur, which is common in small-sized populations which develop in limited spaces.

Current Δ*F* slightly exceeds the recommended maximum of 1%, level below which the fitness of a population steadily decreases^63–65^. Hence, effective population size may still not reach the recommended threshold to maintain genetic variability (≥ 50 individuals). However, the trends described for the historical evolution of Δ*F* report promising outcomes, as there has been a decrease of around 4 points, which may imply genetic variability may be recovering acceptable levels. As Lee and Wilcken^66^ stated, a population of any size can be sustainable if a supplementing source population can effectively suit the required harvest of new individuals and cooperation across institutions is well-established. The incorporation of Accra’s Zoo (within the species geographical range) in 2010, meant an invaluable source for genetically unrelated wild-born animals which may prevent genetic erosion.

The number of equivalent subpopulations below 1 revealed a high level of population structuration. According to Fernández, et al.^67^, population subdivision may be beneficial, provided the extinction risk derived from compromising events such as accidents or health-related factors, may only cause the disappearance of population sections. Furthermore, genetic diversity may reach its highest levels when populations subdivide into as many separate groups as possible. Still, caution should be taken, provided the benefits of subdivision may be counteracted by the negative effects derived from the reduction in effective size and increase in inbreeding.

Although population structure can greatly affect Δ*F*, it hardly affects coancestry increase, hence *N*_*e*_*C*_*i*_ may more accurately estimate effective population size than *N*_*e*_*F*_*i*_^68^. Hence, progressively adding individuals through the participation of new institutions may be beneficial to maintain a high degree of genetic diversity for as long as possible^19^. Simultaneously, the proportion of translocated males is higher than that of females. This practice may be an additional attempt to simulate the male sexual dispersion of the species^60^, an implicit evolutionary strategy for the prevention of inbreeding increase^69–71^.

Values of *f*_*e*_ (15.41) and *f*_*a*_/*f*_*e*_ (0.71) may suggest the frequent use of a small number of animals for breeding may lead to the loss of genetic variability, which may be supported by the low number of ancestors (5) which explains 50% of population's gene pool. Still, founders’ genotypes are represented in the current population. The unequal contribution of founders may be confirmed by the values of *f*_*g*_ (11.85) and *f*_*a*_/*f*_*e*_ (0.71) as one of the main causes for the current genetic diversity loss. The difference between *f*_*e*_ and *f*_*a*_ suggests bottlenecks, although not sharply, may have reduced population’s genetic variability. The lower the *f*_*e*_/*f*_*a*_ is, the greater impact bottlenecks have on the population.

These bottlenecks may be associated to a progressive increase in the occurrence of abnormalities and susceptibility to disease or stressful environmental situations. Such an increased susceptibility may derive from the increase in the incidence of deleterious recessive mutations, which may potentially lead populations to extinction^72^. Mutations that are only mildly deleterious are difficult to eliminate and are the principle cause of inbreeding depression^73^. Furthermore, even if lethal and semi-lethal mutations disappear rapidly due to inbreeding, the large costs of this process may affect population viability^73^.

For instance, infant mortality levels of in white-naped mangabeys in captivity of 37.2% with most deaths occurring within the first two months of life^58^, could potentially be ascribed to increased inbreeding levels (around 25%) in primate captive populations among other factors^74^. However, theoretically, species that are naturally inbred to some degree in the wild, should potentially show less of a deleterious effect when subjected to inbreeding in captivity, which may somehow explain the low representativity of the loss of genetic diversity derived from the occurrence of bottlenecks and genetic drift in the population under study (0.97%)^74^.

Additionally, the difference between *f*_*g*_ and *f*_*a*_ (11) would suggest the effects of genetic drift on genetic diversity may have been compensated by the higher value of *f*_*g*_. The difference between *f*_*g*_ and the number of founders (f) (29) may be indicative of the loss of founder’s offspring, inbreeding increase at founder stages, or a combination of both causes^19,75^. This could be justified by the absence of an intensive population breeding programme until 2000 when the EEP^76^ of this species was set^19^. Nevertheless, according to genetic theory, twenty unrelated individuals may be enough to retain 97.5% of the wild gene diversity within the founder population^72^.

Long generation intervals found in primates may permit genetically self-sustainability with few founders. In fact, this specific ex-situ programme may have effectively captured at least 90% of wild gene diversity for 100 years since the captive population was established, as most of the EEPs and ESBs within EAZA institutions^77^, with levels of genetic diversity (95.78%) in the captive population progressively increasing since 2012 (93%)^19^. Considering that intensive management for this captive population is relatively recent, the maintenance of such high genetic diversity levels may depend on multiple simultaneous factors. For instance, not enough generations may have passed since the decrease in the effectives comprising the wild population pushed the species to its current endangered situation. Hence, generation number in captivity may not be enough to verify the magnitude of genetic variability reduction^8,78^.

However, in the absence of conservation efforts, a substantial loss could be confirmed in the near future for these primates. For instance, as reported by Jara et al.^19^, the number of founder genome equivalents was half the number of founders in captive white-naped mangabeys in 2016, which was indicative of either lost descendants of the original founders or that the original founders were inbred, or a combination of these factors.

In this context, the lower difference between founder genome equivalent and number of founders in the present study confirms special efforts may be being made on the preservation of descendants from the founder population, as if the cause for such differences may have been the fact that founder animals were inbred, these values may have remained somehow stable. This supports the fact that the founder population of captive white-naped mangabeys may have been highly genetically diverse and may have included individuals from a wide range of geographic origins hence, the variability to be expected from wild populations’ sub-structuration may be well represented^79^ as described for other captive populations of critically endangered species^80^.

Genetic diversity may be the basis for individuals’ resilience to the factors that extensively threat their wild populations and the adverse effects of adaptation to captive breeding^81^. In this context, research seeking to understand viability and resilience mechanisms in captive populations may bring about the development of tools which may enable to evaluate genetic diversity levels indirectly. This in turn may help to fulfill conservation purposes more efficiently and practically, as the more diverse populations are, the more capable to adapt to captivity environments they will be as well.

In this sense, a recent population viability analysis simulating different scenarios combining deterministic and stochastic factors and their potential impact on the viability of wild isolated populations of white-naped mangabey has suggested high levels of genetic diversity may be generally maintained under all assumptions (> 90%)^82^. Hence, the subdivided populations could contribute to the conservation of genetic diversity, as shown in wild fragmented populations of other nonhuman primates^83–85^.

The minimum Nei’s genetic distance between institution pairs, effective population size and Wright’s F statistics confirmed a certain subdivision degree. A single institution (26) is at the top of the relationship cladogram (Fig. 5). This may base on the private character of the institution and on the frequent translocation of the offspring born to other zoos, while no genetic material is received (from live animals or assisted reproduction).

Reproductive policies normally consider a small number of ancestors as the basis for subsequent generations, which indirectly replicates the natural isolation patterns found for fragmented wild animal populations^86^. Table 5 suggests the breeding strategy should aim at mating animals keeping relationship coefficients (R) below 10% to maintain the inbreeding below 1%, which may increase effective population size up to a minimum of 50 to counteract the risk of extinction. Breeding animal selection should consider conservation criteria such as mean kinship rankings (average relatedness value of an animal towards the current population) to reduce inbreeding and genetic variability loss^87^.

Bearing this in mind, current pairing/transfer criteria focuses on ranking animals in the population considering their individual inbreeding coefficients (F) and genetic conservation index (GCI). GCI^88^ measures the proportion of genes of founder animal *i* in the pedigree of each particular individual in the population. GCI is a measure of the representativity of founding population in the individuals, and acts as a measure of genetic diversity in the range of the genetic pool of the base population. The highest score in the rank was given to the model obtaining the most desirable value for each particular criterion. For instance, those individuals presenting the lowest inbreeding coefficients may be ranked higher, while those animals presenting the highest genetic conservation indexes will be ranked higher as well. Then, the rest of positions in the rank were determined in ascending or ascending order from the most desirable values to the lowest desirable ones, which are ranked with the value of 1.

Afterwards, as aforementioned inbreeding and GCI differ in terms of which their most desirable values are and what their magnitude is, a combined selection index (ICO) is developed following the premises in Van Vleck^89^ to summarize the position in the rank for each of the two parameters. The combined index used (ICO) was as follows;

$$ICO=\frac{Inbreeding \, coefficient  \, Position \,  in  \, the \,  Rank  \, * \, \mathrm{W}1+ GCI  \, position  \, in  \, the  \, Rank \, * \, \mathrm{W}2}{2}$$

where W_1_ is the weight for inbreeding coefficient, W_2_ for GCI rank position. All criteria are given the same relevance in the ICO, hence, no coefficient was used, that is the proportion of 1:1 is followed. As a result, the animals presenting greater ICO values are the ones presenting the highest levels of genetic diversity from the pool of the founding population and having those founding genes from the least related animals. Conclusively, the individual values for ICO and mean kinship rankings between pairs of individuals are considered to determine which the most appropriate pairing/transfer candidates are.

This use of mean kinship, inbreeding and GCI values for best guiding of animal pairing is proposed to be more attainable in zoo-kept intensive-managed populations than in other large housing facilities where social structure and therefore mate choices cannot be accurately handled^4^. Such condition may translate into a more successful genetic and demographic intensive management of biodiversity conservation^43^. Comparing genetic diversity and structure between captive and wild populations using genomic markers would help to determine the magnitude of the potentially occurring bias when using information from pedigrees and which of these two alternatives may eventually more effective^26,90^.

## Methods

### White-naped mangabey breeding-management programme in captivity

Barcelona Zoo coordinates the European Studbook (ESB) for the white-naped mangabey. This population breeding/management programme registers the information in respect birth place, birth and death dates, average kinship (average relatedness between an individual to all others in the population, including itself) and transfers of the individuals that are housed in EAZA-member institutions.

Through the compilation of this information, a demographic and genetic assessment is regularly performed to effectively manage the general status of the population. If derived results indicate a non-self-sustaining population at a given time, more intensive management (i.e. increasing the rate of exchange of individuals between zoos and planning matings carefully considering their diversity, inbreeding levels and relatedness) are proposed for the ongoing population viability^19^.

Transfer decisions are based on internal criteria such as lack of space for more individuals in a particular location for welfare issues, existing heavy disputes among congeners sharing resources, desired phenotypic traits and/or low reproduction performance within a captive herd.

Concerning exchanging rates, sixty-eight males and fifty-seven females have been subjected to translocation activities for improved pairing since 1994. In fifty-five cases, this genetic exchange has been made through assisted reproduction techniques by expert veterinarians instead of removing the animals from their living emplacement for mating attending to animal welfare-related logistic and biological constraints (transport and potential destabilization of social hierarchy in acceptor herd).

### Data registries and software tools

The historical population comprises 298 animals (157 males and 141 females) born between January 1951 and January 2019. The current population comprised 120 white-naped mangabeys (54 males and 66 females) which were born between September 1987 and January 2019 and are alive. Only thirty-three animals (11%) were wild-born.

Thirty-four European Association of Zoos and Aquaria’s member zoos houses white-naped mangabeys and compiles genealogical information for the commitment of this species conservation program’s goals^19^ (Fig. 1). The studbook was provided by the white-naped mangabey EEP coordinator. The registries consist of the individual name and identification code, sire code, dam code, sex, birthdate (to know the temporal evolution or tendency of some parameters), birthplace (captive-born or wild-born) and status (death or alive).

The demographic and genetic parameters of variability were evaluated using the ENDOG software (v 4.8)^91^. The analysis of the probabilities of genetic origin and ancestral contributions was carried out with the CFC software^92^, on all the data sets. Dendroscope 3 software^93^ was used for the graphical representation of the dendrogram based on Nei's genetic distance between subpopulations.

### New-born annual increase and pedigree completeness index

New-born annual median number, maximum and mean number of offspring per sire and dam were calculated. Pedigree completeness index (PCI), which summarizes the percentage of known ancestors of each ascending generation, was evaluated as in Navas et al.^62^ computing the maximum number of traced generations; the number of complete traced generations; the number of complete equivalent generations (all known ancestors); and the quality of the genealogical information of the pedigree were determined after the calculation of the proportion of known parents through to great-great-great-great-grandparents (first to fifth generation inclusive).

### Breeding animals, generation interval and mean age of parents at offspring’s birth

Generation intervals were computed as the mean age of parents at the birthdate of their offspring selected for breeding^94^ and the mean age of parents at offspring’s birth (selected for breeding or not), were calculated for each of the four gametic pathways: sire to son, sire to daughter, dam to son and dam to daughter. These parameters were obtained from the birthdate for every animal together with those of its parents. Female/male ratio was considered the relationship between total number of females and males in historical and current populations.

### Identity by descent estimators and degree of non-random mating

Individual inbreeding (*F*) was computed according to Luo^95^. The average relatedness (Δ*R*) of each individual or the probability that an allele randomly selected within the population belongs to a given animal, was obtained as proposed by Gutiérrez et al.^91^. The individual rate of inbreeding ($\Delta F)$ for the number of complete equivalent generations was computed according to Gutiérrez et al.^96^. The individual rate of coancestry ($\Delta C)$ for the number of complete equivalent generations was computed as suggested by Cervantes et al.^97^. Mean inbreeding (F) per generation and average relatedness (Δ*R*) were used to issue regression equations fitting lineal, logarithmic and polynomic functions to predict for the evolution of inbreeding and relatedness up to fifteen generations onwards. Non-random mating (*α*) was calculated as described by Caballero and Toro^98^. Genetic Conservation Index (GCI) or the effective number of founder ancestors of each pedigree, was estimated as proposed by Alderson^88^.

### Probabilities of gene origin, ancestral contributions and genetic diversity

The effective number of founders (*f*_*e*_) or founders equally contributing that are expected to generate the same genetic diversity that in the studied population, was computed as;

$${f}_{e}=\frac{1}{\sum_{k=1}^{f}{q}_{k}^{2}}$$

where *q*_*k*_ is the probability of gene origin of the founder and *f* the real number of founders^75^.

The minimum number of ancestors (*f*_*a*_), founders or not, necessary to explain the entire genetic constitution of the population, was determined as;

$${f}_{a}=\frac{1}{\sum_{k=1}^{f}{p}_{k}^{2}}$$

where *p*_*k*_ is the marginal contribution of an ancestor *k*, which means the contribution not explained yet by the rest of ancestors^99^. Both parameters (*f*_*e*_ and *f*_*a*_) can be used to summarise the loss of genetic variability because of the non-proportional breeding animals’ contribution^100^.

The effective number of founder genomes (*f*_*g*_) or the number of equally contributing founders without founder alleles loss that are expected to generate the same genetic diversity than in reference population (both parents known), was obtained by calculating the inverse of twice the average coancestry^98^.

The expected marginal contribution of each major ancestor *j* (the largest genetic contributing founders or not) was computed as the expected genetic contribution independently of the rest of ancestors’ contribution^99^.

The contributions to inbreeding of nodal common ancestors (highest marginal genetic contributions) that form inbreeding loops, were obtained according to Colleau and Sargolzaei^101^. An inbreeding loop exists when the ancestor of an individual is that by both maternal and paternal pathway. Mean effective population sizes ($\overline{{N}_{e}}$)^102^, was calculated as;

$$\overline{{N}_{e}}=\frac{1}{\left(2\overline{\Delta IBD}\right)}$$

The number of equivalent subpopulations^103^ was assessed as the relationship between $\overline{{N}_{e}Ci}=\frac{1}{\left(2\overline{\Delta C}\right)}$ or the mean effective population size considering the coancestry coefficient and $\overline{{N}_{e}Fi}=\frac{1}{\left(2\overline{\Delta F}\right)}$ that is the mean effective population size considering the inbreeding coefficient. Genetic diversity (GD) was calculated as^75,104^;

$$GD=1-\frac{1}{{2f}_{g}}$$

The GD loss (GDL) in the population since the founder generation was estimated as $1-GD$. Considering the different possible causes of this loss, GDL derived from the unequal contribution of founders was calculated as;

$$\mathrm{GDL from the unequal contribution of founders }=1-{GD}^{*}$$

where^98^,

$${GD}^{*}=1-\frac{1}{{2f}_{e}}$$

The difference between GD and GD* is referred to genetic drift accumulated since the foundation of the population^75^.

The effective number of non-founders (*N*_*ef*_) was calculated as proposed by Caballero and Toro^98^ to describe the relationship between the effective number of founders and the number of equivalent genomes of founders.

### Zoo relationships and breeding strategy

The relationships between zoos were evaluated using Wright’s F statistics and Nei’s genetic distance. The Wright’s F statistics^105^ for each subpopulation (35) were calculated according to Caballero and Toro^106^. Wright’s F statistics allow pairwise comparisons among subpopulations or populations but those pairwise "distances" take account only of the data for the two populations concerned, not all the data simultaneously. Still this provides relevant information in the context of pedigree evaluation as the differences between both parameters may account for the estimation bias that may occur. For this reason and to quantify the degree to which populations differs from the entire pool of data using distance measures that make biological assumptions, Nei’s distances were used as well. Nei’s genetic distance^69^ between subpopulations *i* and *j* was computed as;

$$ {\text{D}}ij = \left[ {\left( {{\text{C}}ii + {\text{C}}jj} \right)/{2}} \right] - {\text{C}}ij, $$

where C*ij* is the average pairwise coancestry between individuals of the subpopulations *i* and *j*, including all N*i* × N*j* pairs. C*ii* and C*ij* are the average pairwise within subpopulations *i* and *j*, to assess interzoo relationships.

Afterward, a simulation was made to determine the maximum limit of relatedness coefficient existing in the population between mated animals to determine which matings maintained ($\Delta F$) in a generation equal or below 1%.

These levels of individual increase in inbreeding correspond to *N*_*e*_ = 50. Below these levels fitness of a population noteworthily decreases^107^. Relatedness coefficient (Δ*R*) can be defined as the probability that two individuals share an allele because of common ancestry. Relatedness coefficient (Δ*R*) of a pair of mating animals is the potential inbreeding coefficient of their potential offspring. This parameter ranges from 0 (unrelated) to 1 (clones or identical twins). This definition excludes alleles that are shared because of belonging to the same species or population.

Five mating groups were considered for the simulation. The average relatedness coefficient between mated animals was kept below 0.00%, 5.00%, 10.00%, 15.00% and 20.00% (greatest feasible limit considering all possible mating among all 120 alive animals). The inbreeding coefficient of the offspring for each mating was estimated as one-half of the parental relationship coefficient. The inbreeding rate^96^ was calculated by averaging the individual inbreeding increase through;

$$\Delta Fi=1-\sqrt[ti-1]{1-Fi}$$

where *t*_*i*_ is the number of complete equivalent generations^108^ and *F*_*i*_ the inbreeding coefficient of the individual *i*.

For each group, 17 random matings were selected, basing on the number of births in the last natural complete year (2018: 17 births) and on the assumption of one baby per female^42^ using SPSS Inc.^109^. Thirty replicates were evaluated within each group to calculate the average effective population size (*N*_*e*_) as described by Gutiérrez et al.^96^.

## Supplementary Information

Supplementary Information.

## Data Availability

The datasets generated during and/or analyzed during the current study are available from the corresponding author on reasonable request.
